# Supplementary material for: Tribo-electrochemistry induced artificial solid electrolyte interface by self-catalysis
Source: Nat Commun. 2021 Dec 10;12:7184. doi: 10.1038/s41467-021-27494-z (PMC8664887; doi:10.1038/s41467-021-27494-z)
Supplement: Supplementary file 1 — Supplementary Information file [file 41467_2021_27494_MOESM1_ESM.pdf]

Supplementary Information

Tribo-electrochemistry induced Artificial Solid Electrolyte Interface by Self-catalysis

Chichu Qin [1, 2], Dong Wang [1, 2], Yumin Liu [1], Pengkun Yang [1], Tian Xie [1], Lu Huang [1, *], Haiyan Zou [1], Guanwu Li [1], Yingpeng Wu [1, *]

1 State Key Laboratory of Chem/Bio-Sensing and Chemometrics, Advanced Catalytic Engineering Research Center of the Ministry of Education, College of Chemistry and Chemical Engineering, Hunan University, Changsha, 410082, P. R. China.

2 These authors contributed equally: Chichu Qin, Dong Wang

**Correspondence: luhuang@hnu.edu.cn (L. Huang); wuyingpeng@hnu.edu.cn (Y. P. Wu)*

Supplemental Figures and Supplemental Tables.

Supplementary Figures:

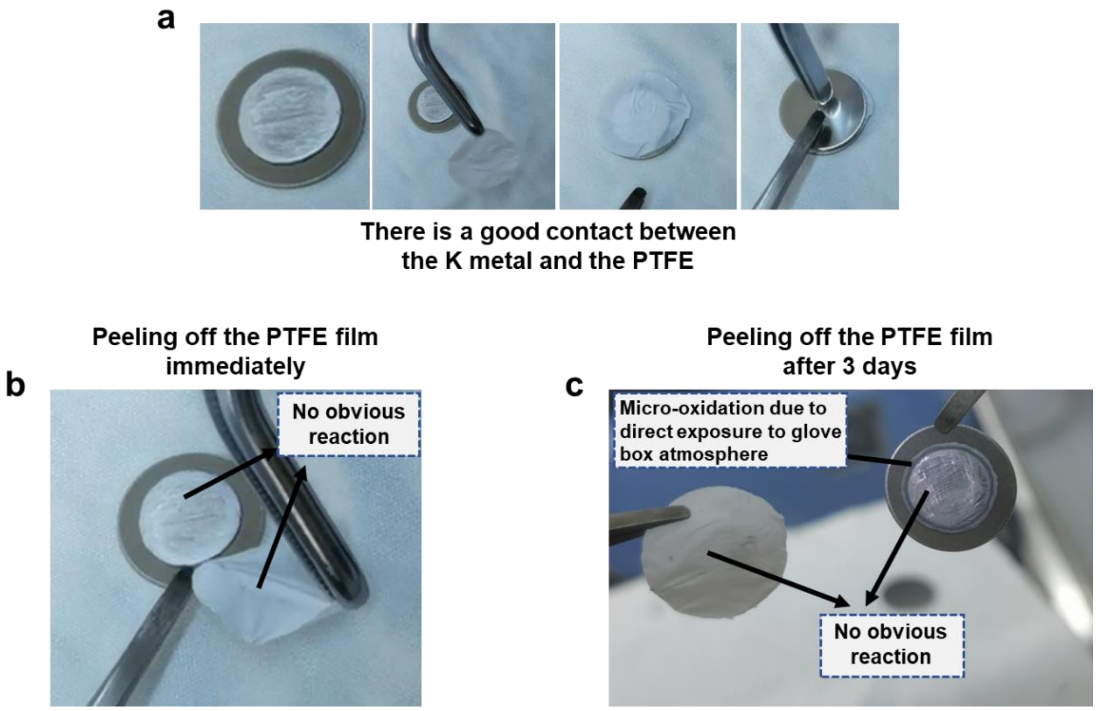

Fig. S1 Phenomena that there is no obvious reaction in direct contact between solid K and PTFE. (a) Optical photographs of good contact between PTFE film and K metal with surface oxide scraped off. (b) Immediately peeling off the PTFE film after a good contact between PTFE and K metal, there was no significant reaction between PTFE and K metal. (c) Peeling off the PTFE film after 3 days, there was also no significant reaction between PTFE and K metal.

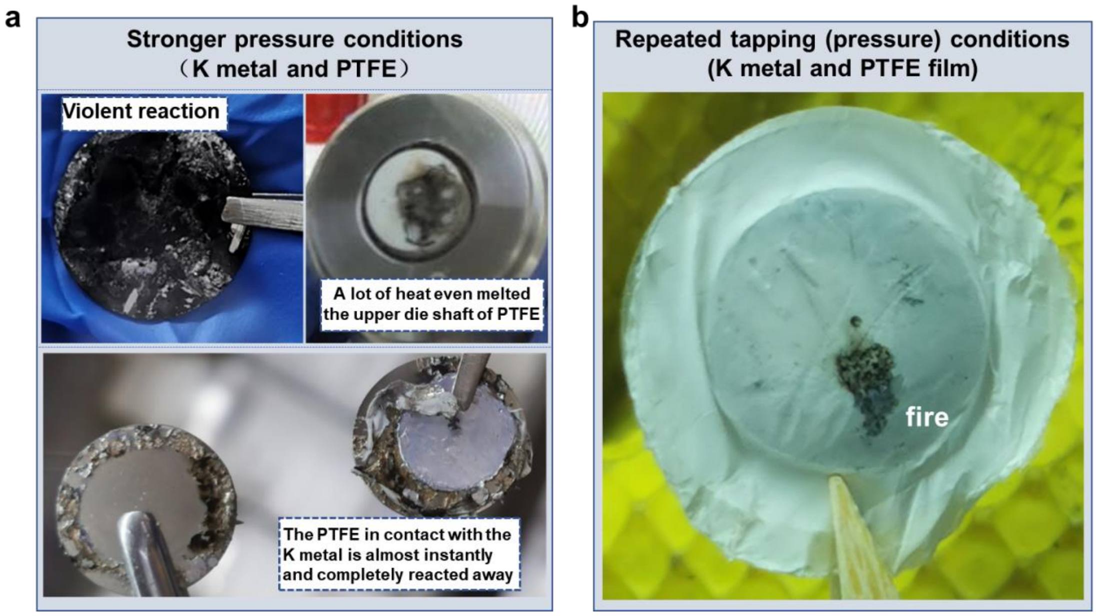

Fig. S2 Phenomena that there are rapid and violent reactions when a force applied to the joint surface of solid K and PTFE. (a) A compressive stress is applied between K metal and PTFE. The reaction between PTFE and K metal is very violent at once and produces a black material. The generated heat can even melt and corrode the upper die shaft, which is made of PTFE. (b) Rubbing on the PTFE film, which is in close contact with the K metal underneath, can cause a violent reaction and even fire.

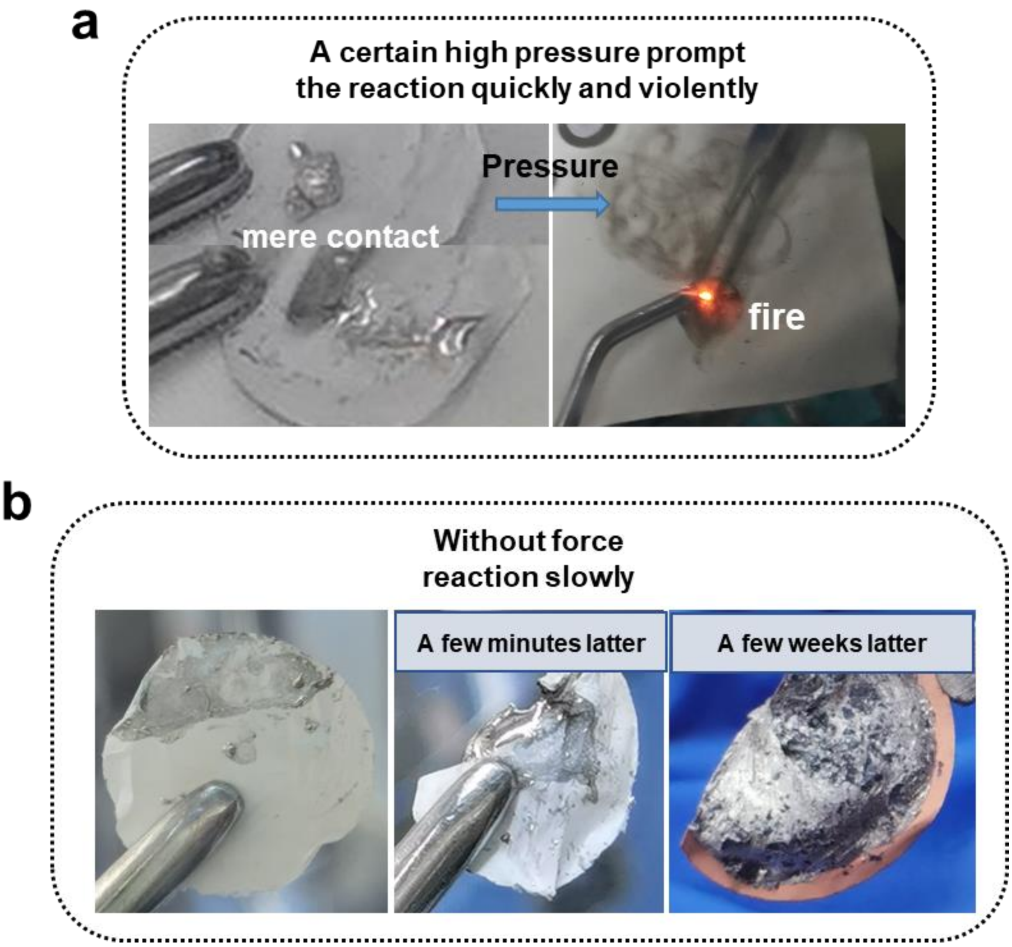

Fig. S3 Different reaction phenomena between liquid Na-K alloy and PTFE with or without external force. (a) It is difficult to react rapidly and violently in the absence of any other external force when the direct contact between liquid alkali metal (Na-K alloy) and PTFE film (left). If a compressive stress is applied between the two, there is a drastic action and even a fire (right). (b) Liquid Na-K alloy reacts slowly when only direct contact with PTFE film.

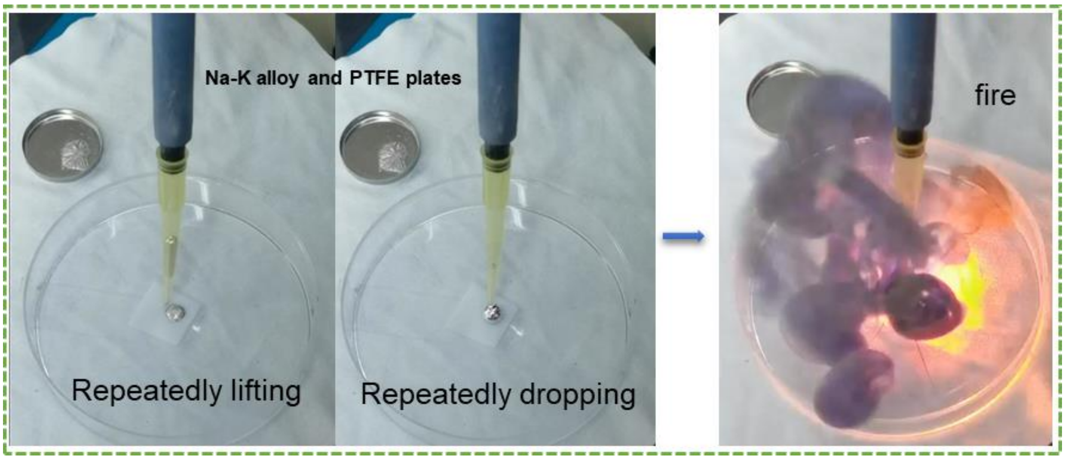

Fig. S4 Fire phenomenon when repeatedly lifting and dropping the liquid Na-K alloy droplet onto the PTFE plate.

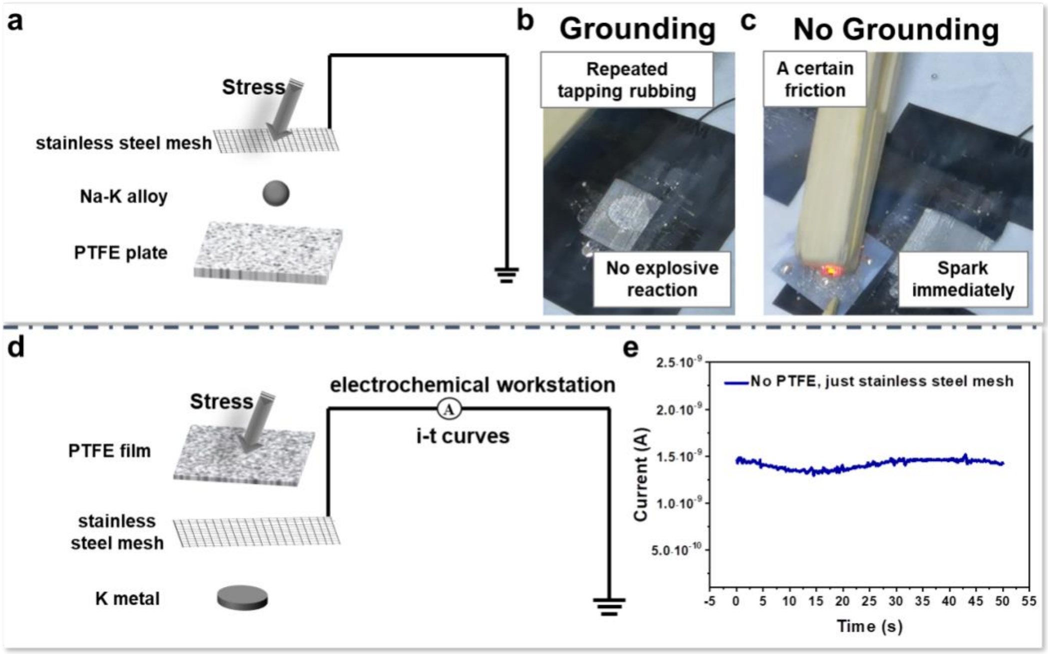

Fig. S5 Charge measurements by using the grounding device. (a) Schematic diagram of the grounding device for the charge export. (b) Though repeat tapping and rubbing, there is no explosive reaction between PTFE plates and Na-K liquid alloy when grounding. (c) There will be an immediate spark even if only a little friction is applied once out of the grounding device. (d) Schematic diagram of the grounding device for the charge export and i-t curves tests. (e) Pressure applied regularly every 5 s as in Figure 1 c without PTFE and there is no special regular current signal. Source data are provided as a Source Data file.

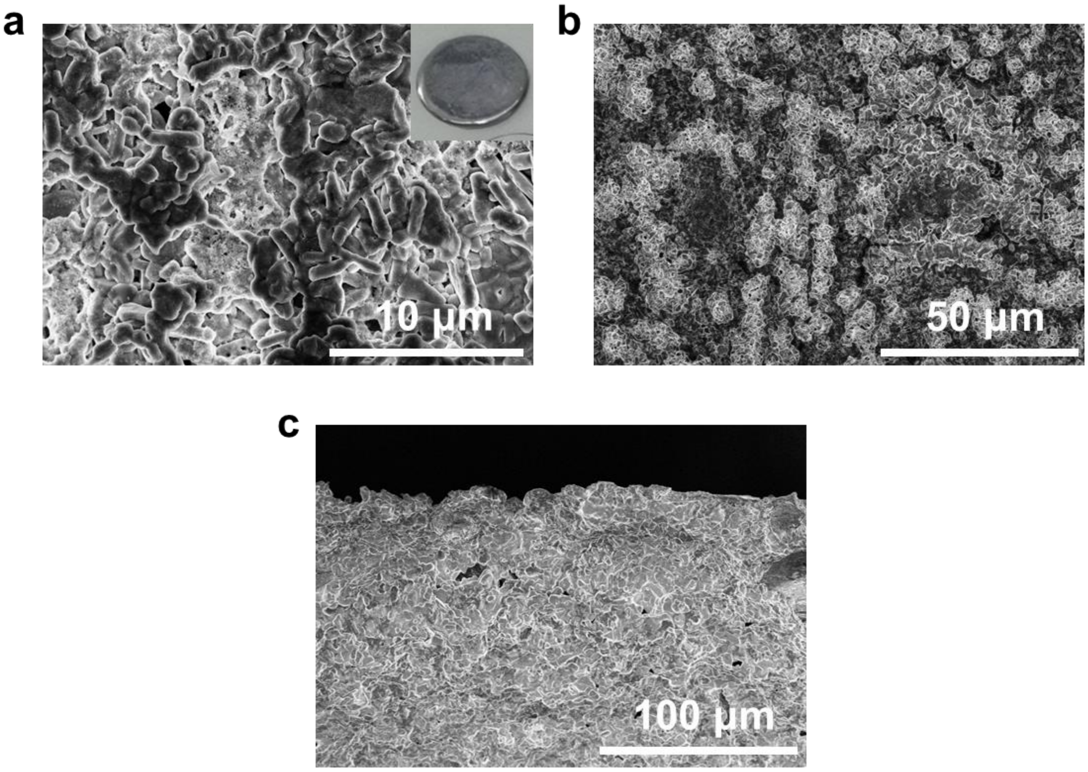

Fig. S6 SEM images of pristine K. (a, b) Top views of pristine K, the illustration in (a) is an optical photograph of pristine K anode. (c) Cross-sectional view of pristine K**.**

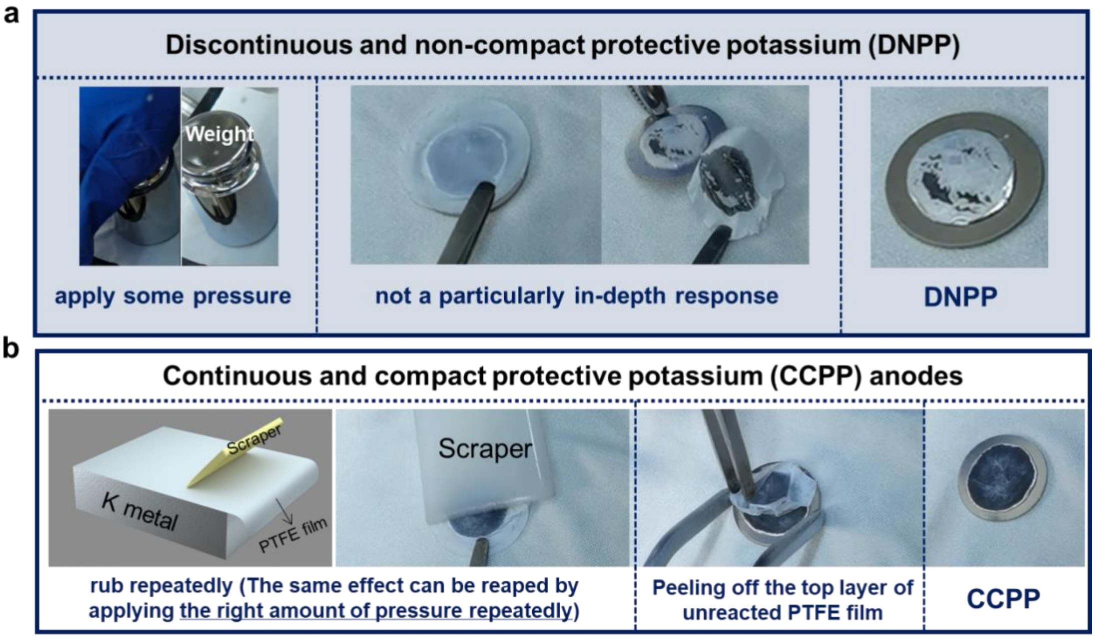

Fig. S7 Schematic diagrams of DNPP and CCPP samples' preparation. (a) If statistic compressive stress is applied to the joint surface of PTFE and K metal, a discontinuous and non-compact SEI will generated on the surface of the potassium metal due to the unevenness of the surface of the potassium metal and the insufficient depth of the reaction, we call it discontinuous and non-compact protective potassium (DNPP). (b) Schematic diagram of CCPP sample's preparation. Different from DNPP, if the force work repeatedly, the negative charge on the PTFE will generated evenly and guide the continuous and even tribo-electrochemistry reaction. As a result, a complete and compact protective layer is formed, we call it continuous and compact protective potassium (CCPP).

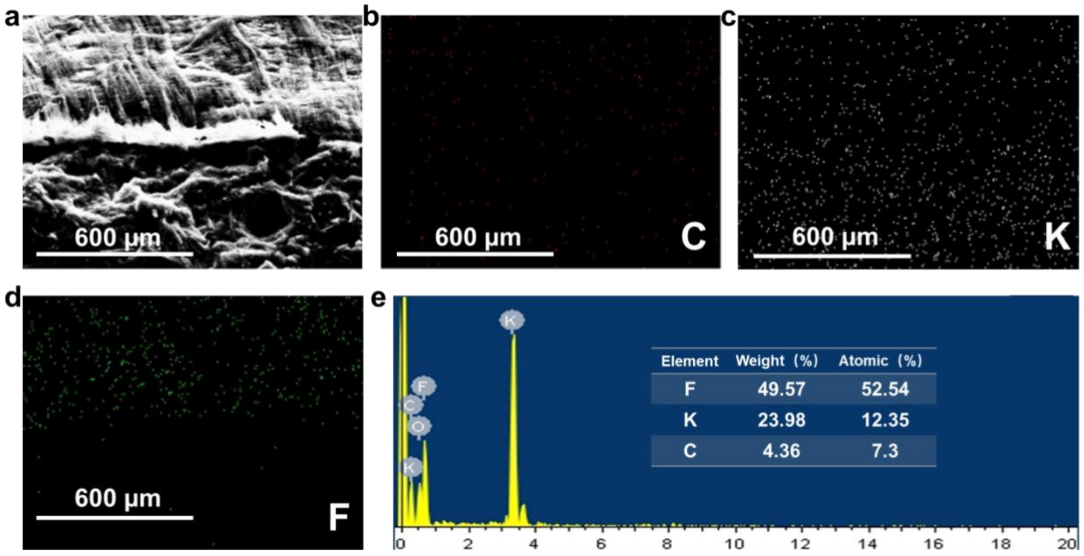

Fig. S8 The elemental mappings of the CCPP in the sectional view. Source data are provided as a Source Data file.

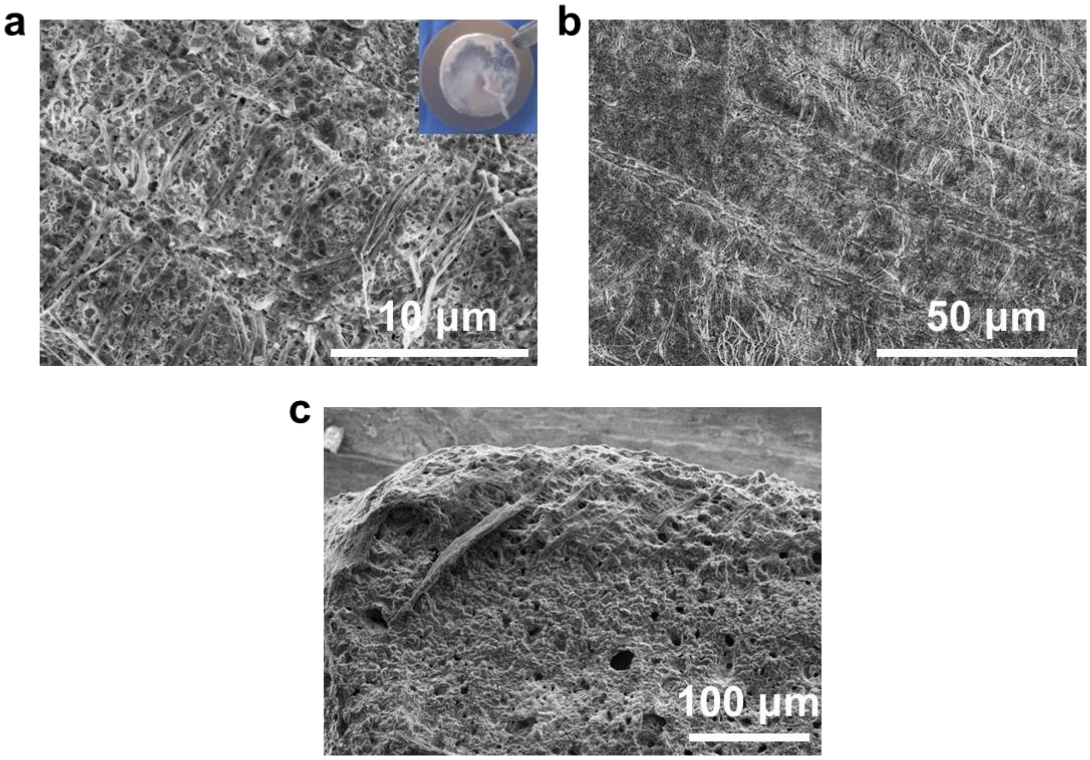

Fig. S9 SEM images of DNPP. (a, b) Top views of DNPP, the illustration in (a) is an optical photograph of DNPP. (c) Cross-sectional view of DNPP.

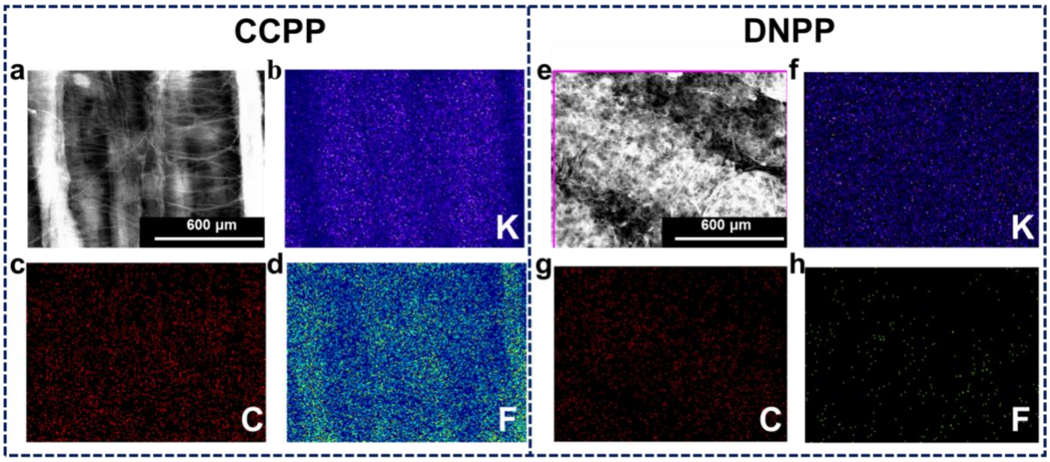

Fig. S10 The elemental mapping contrast of CCPP and DNPP under the same analytical conditions. (a-d) Elemental mappings of CCPP. (e-h) Elemental mappings of DNPP. Source data are provided as a Source Data file.

Herein, it can be clearly seen that the distribution of the SEI of CCPP is uniform and continuous, while the distribution of the SEI of DNPP is discontinuous and incomplete.

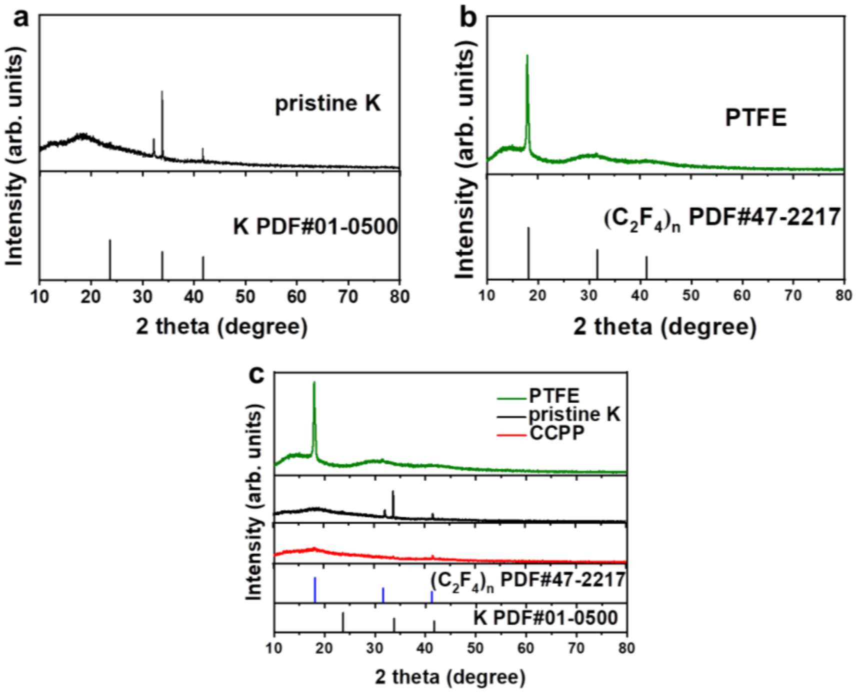

Fig. S11 XRD patterns of CCPP, pristine K and PTFE. (a) XRD pattern of pristine K. (b) XRD pattern PTFE. (c) XRD contrast patterns of pristine K, PTFE and CCPP in the same intensity range. Source data are provided as a Source Data file.

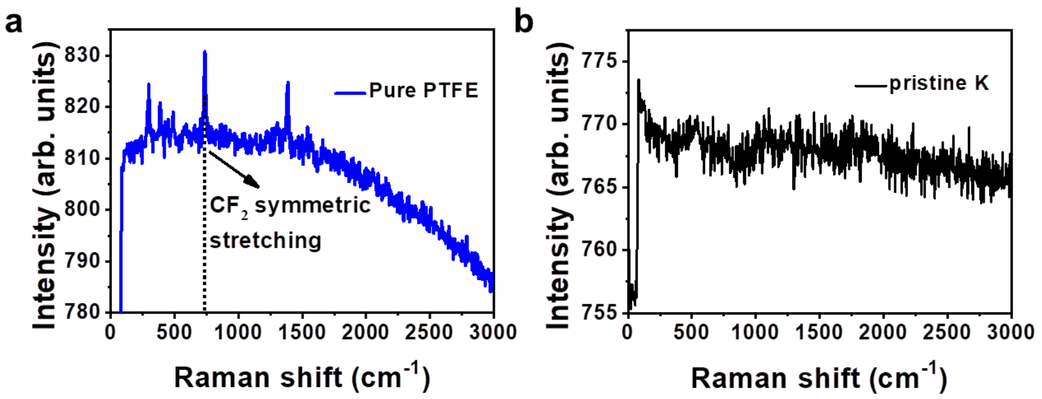

Fig. S12 Raman spectra of (a) Pure PTFE and (b) pristine K. Source data are provided as a Source Data file.

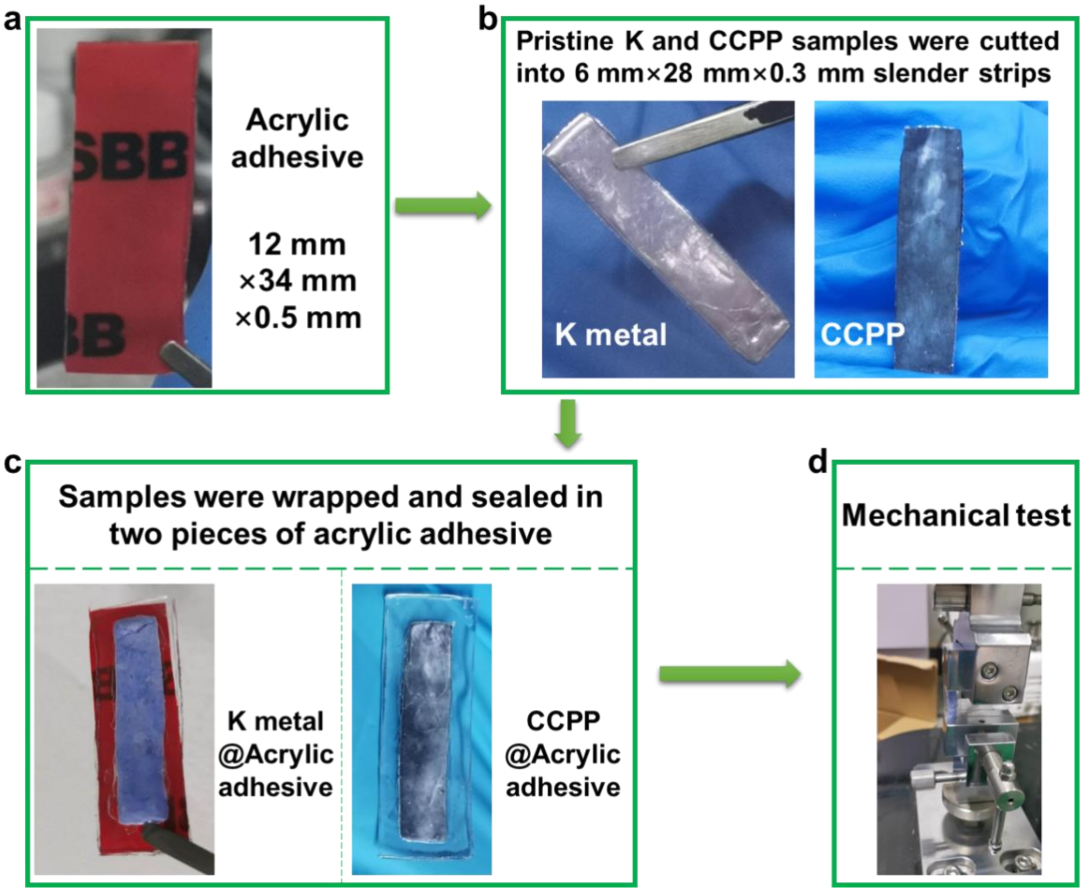

Fig. S13 Mechanical testing process. (a-c) Preparation of sample strips. (d) Mechanical test process.

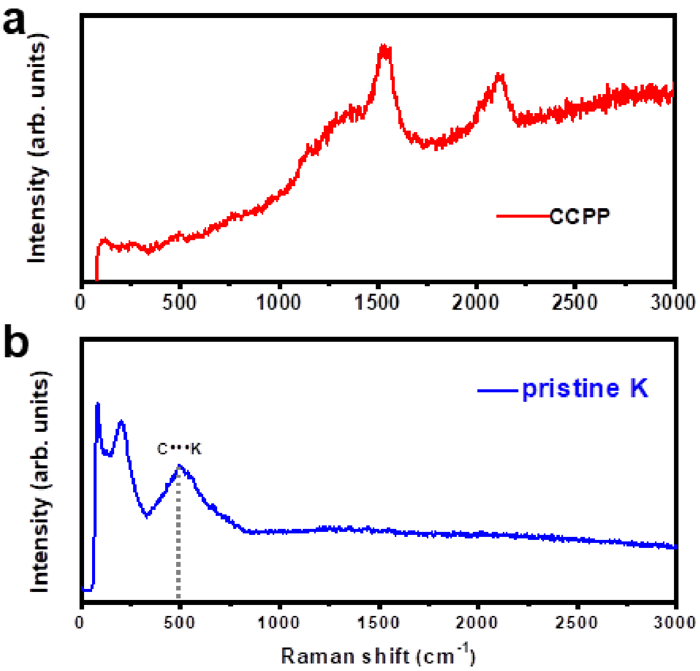

Fig. S14 Raman spectra of CCPP and pristine K after cycling. (a) Raman spectrum of CCPP after first-cycle deposition process. (b) Raman spectrum of pristine K after first-cycle deposition process. Source data are provided as a Source Data file.

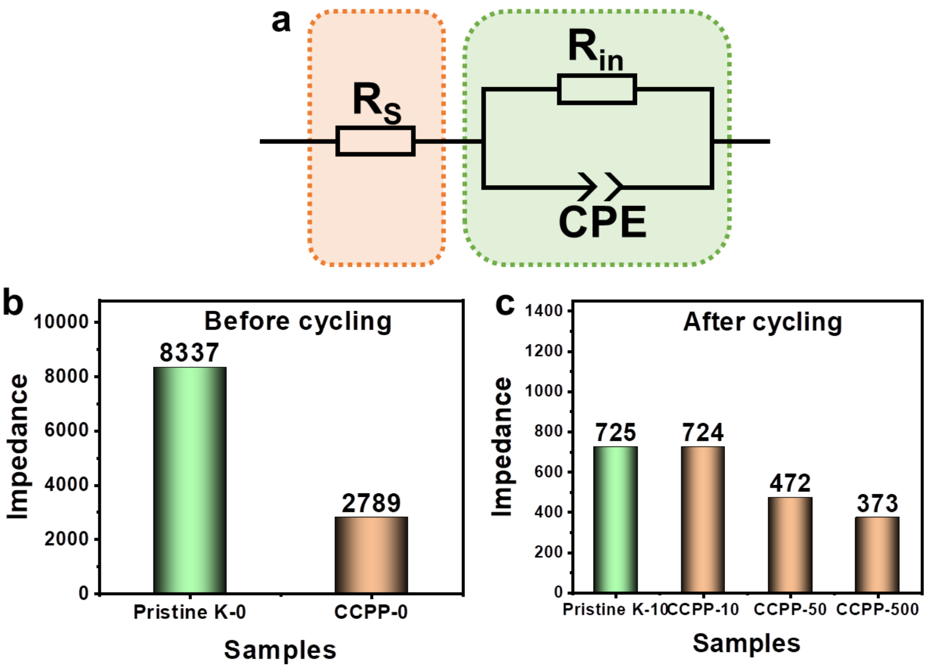

Fig. S15 The equivalent circuit and calculated interface resistance (R_{in}) of pristine K and CCPP. (a) The equivalent circuit of pristine K and CCPP. (b) The calculated interface resistance (R_{in}) of the two EIS date before cycling. (c) The calculated interface resistance (R_{in}) of the two EIS date after cycling. Source data are provided as a Source Data file.

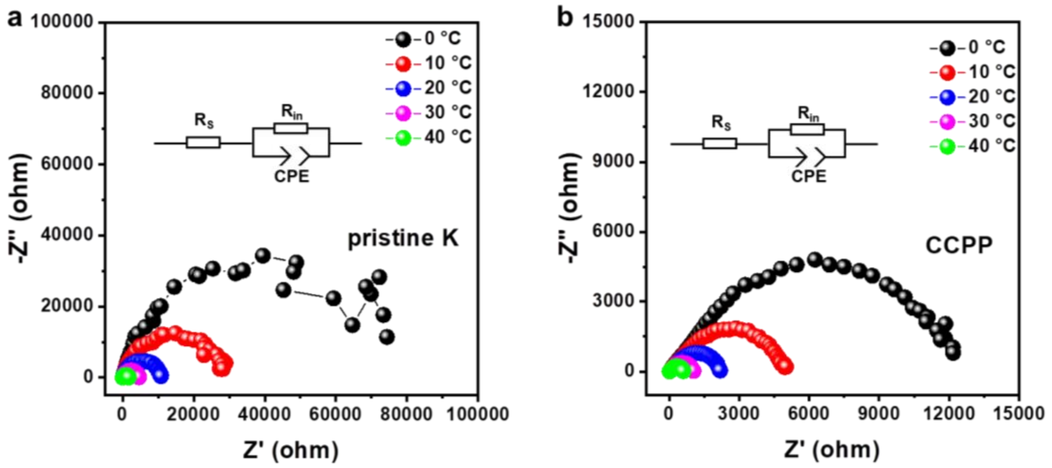

Fig. S16 EIS measurements at different temperatures for (a) pristine K and (b) CCPP. Source data are provided as a Source Data file.

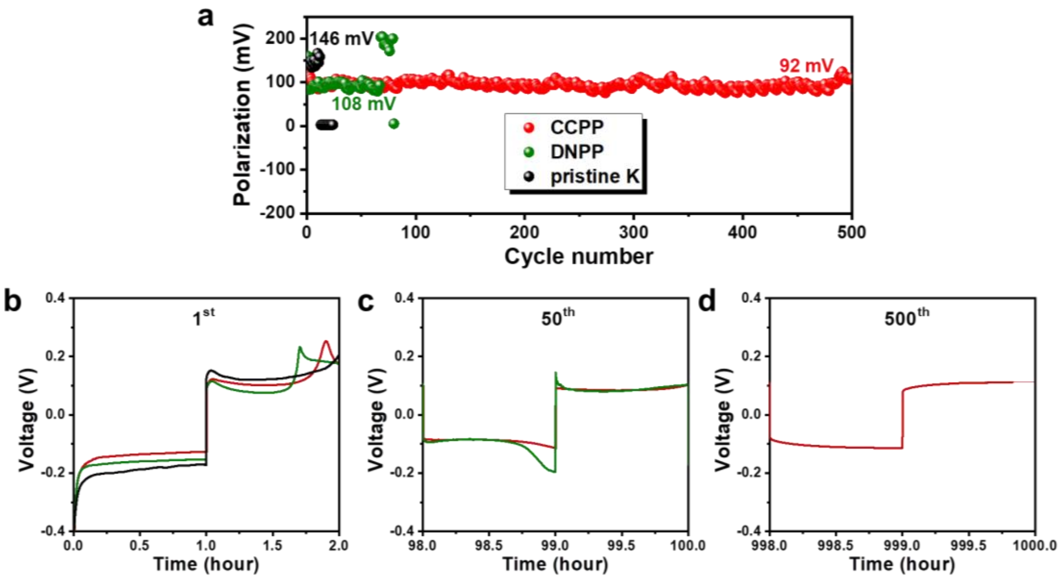

Fig. S17 The polarization voltages and detailed comparisons of CCPP, DNPP and pristine K at 0.5 mA cm^{-2} and 0.5 mAh cm^{-2} in the highly concentrated KFSI/EC:DEC electrolyte. (a) The polarization voltages of CCPP, DNPP and pristine K. (b-d) The detailed comparisons of these three at 1st, 50th and 500th, respectively. Source data are provided as a Source Data file.

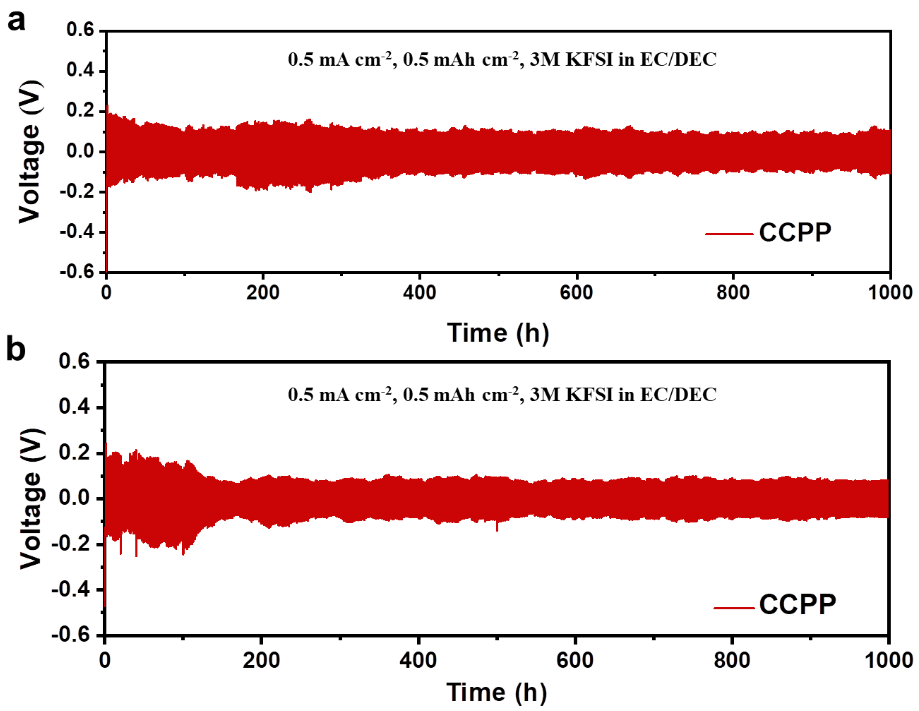

Fig. S18 (a, b) Voltage-time profiles of CCPP at the current density of 0.5 mA cm^{-2} and capacity of 0.5 mAh cm^{-2} in the highly concentrated KFSI/EC:DEC electrolyte. The repeated data related to Figure 4a. Source data are provided as a Source Data file.

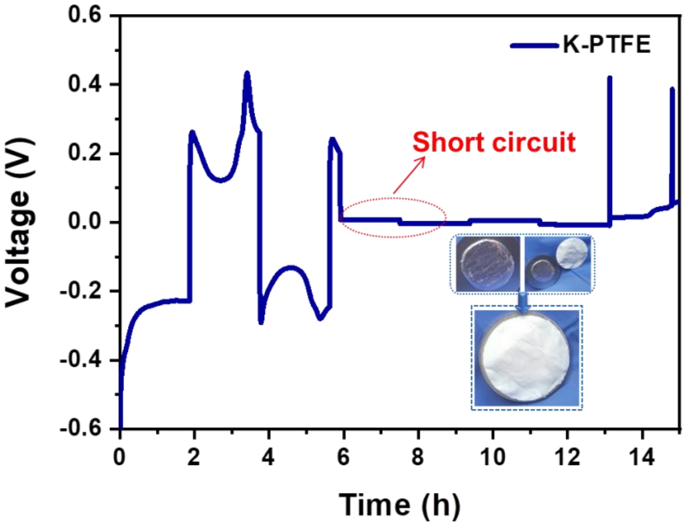

Fig. S19 Voltage-time profiles of the electrode by directly covering a single layer of PTFE on the K metal surface at the current density of 0.5 mA cm^{-2} and capacity of 0.5 mAh cm^{-2} using highly concentrated KFSI/EC:DEC electrolyte. Source data are provided as a Source Data file.

Herein, the electrode quickly failed due to a short circuit. It further shows that the unreacted PTFE on the protective layer has no contribution to the excellent performance.

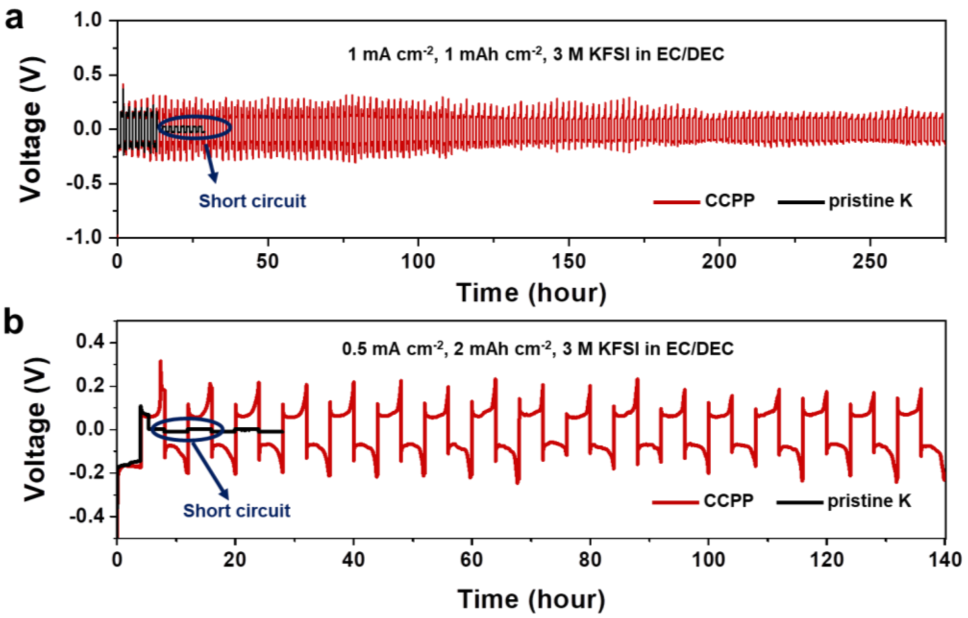

Fig. S20 Voltage-time profiles of CCPP and pristine K at the (a) current density of 1 mA cm^{-2} and capacity of 1 mAh cm^{-2}, (b) current density of 0.5 mA cm^{-2} and capacity of 2 mAh cm^{-2}. Source data are provided as a Source Data file.

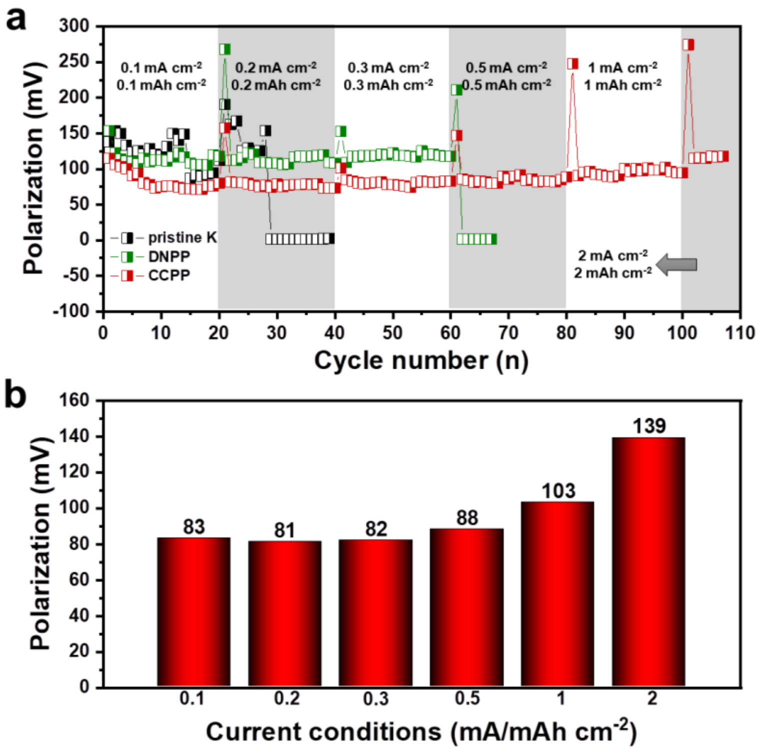

Fig. S21 (a) The polarization voltages and (b) the average polarization voltages of CCPP, DNPP and pristine K at different current and capacity conditions. Source data are provided as a Source Data file.

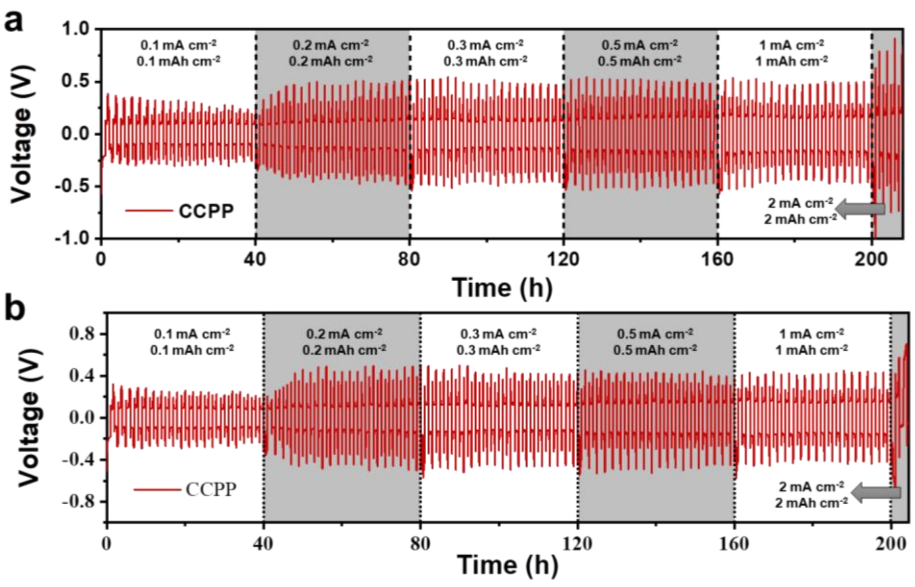

Fig. S22 (a, b) Rate performances of the CCPP anode at different current densities. The repeated data related to Figure 4b. Source data are provided as a Source Data file.

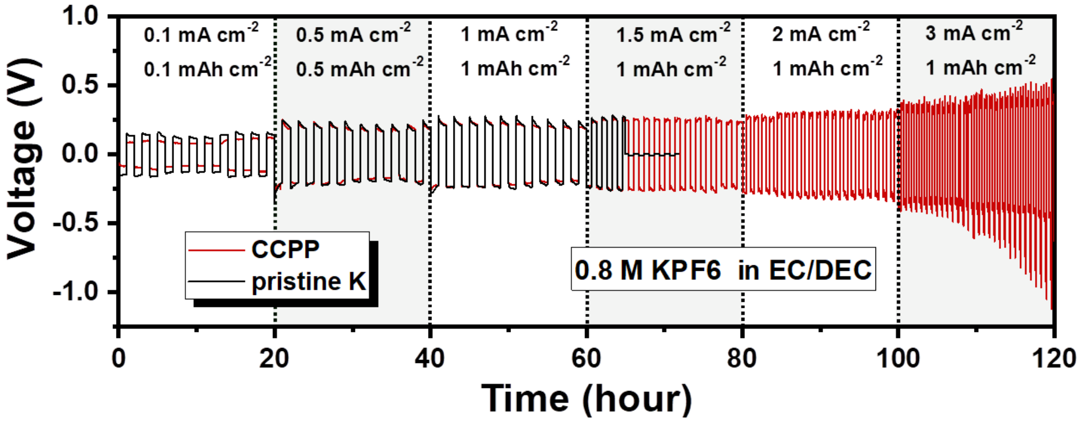

Fig. S23 Rate performances of CCPP and pristine K at different current densities in the KPF_6/EC:DEC electrolyte. Source data are provided as a Source Data file.

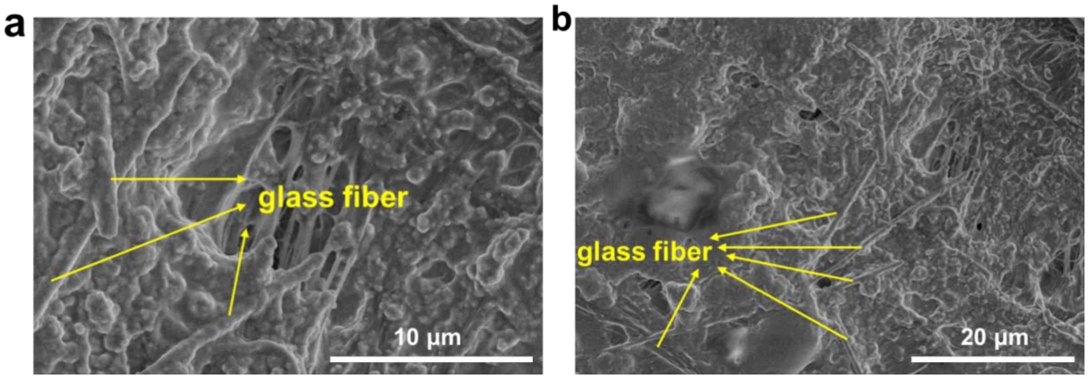

Fig. S24 SEM images of the CCPP anode after 1000-h symmetric cycling at 0.5 mA cm^{-2} and 0.5 mAh cm^{-2}. Only one layer of glass-fiber separator is used here.

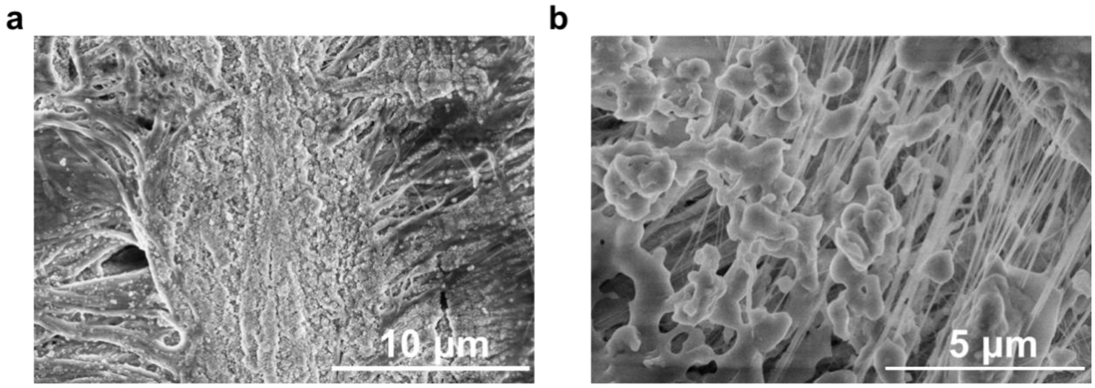

Fig. S25 Top view SEM images of CCPP anode after (a) 10 and (b) 50 cycles.

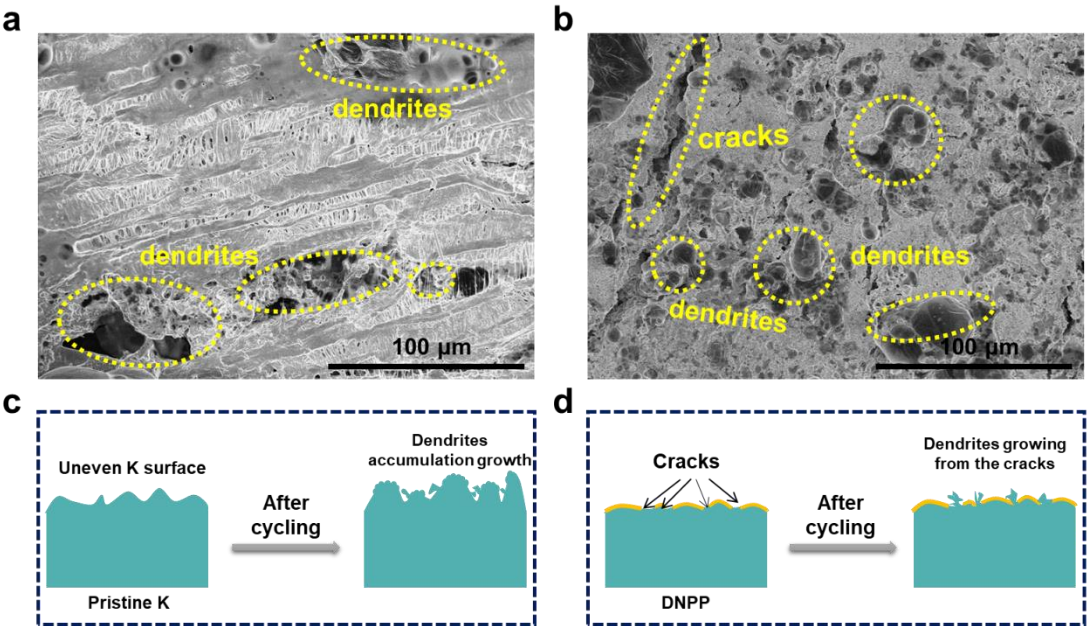

Fig. S26 (a, b) Top view SEM images of DNPP anode after (a) 10 and (b) 50 cycles. (c) Dendrites formation diagram of pristine K. (d) Dendrites formation diagram of DNPP.

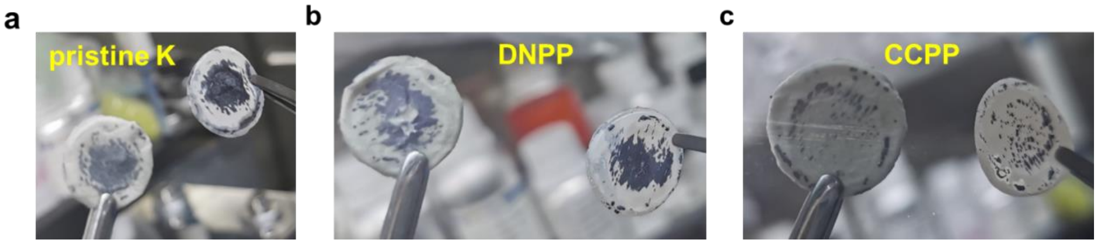

Fig. S27 Optical photographs of the separator on the plating side after 100-h symmetric cycling at 0.5 mA cm^{-2} and 0.5 mAh cm^{-2} for (a) pristine K, (b) DNPP and (c) CCPP anode, respectively. The electrolyte used is 3 M KFSI in EC/EDC (1:1 vol%), a layer of Celgard 2325 is placed between the plating side anode and the glass-fiber diaphragm.

Herein, it can be seen from the state of the diaphragm on the plating side of CCPP, DNPP, and pristine K after 100-hour symmetrical cycling. The growth of dendrites on pristine K in the symmetrical cells were very severe and spread deeply into the separator, causing significant damage (Figure S27a). DNPP is a little better than pristine K, but the SEI on its surface is not dense and thick enough to suppress dendrite growth, so the dendrite still penetrates the separator to some extent (Figure S27b). CCPP maintains excellent diaphragm integrity even after 100-h of cycling (Figure S27c).

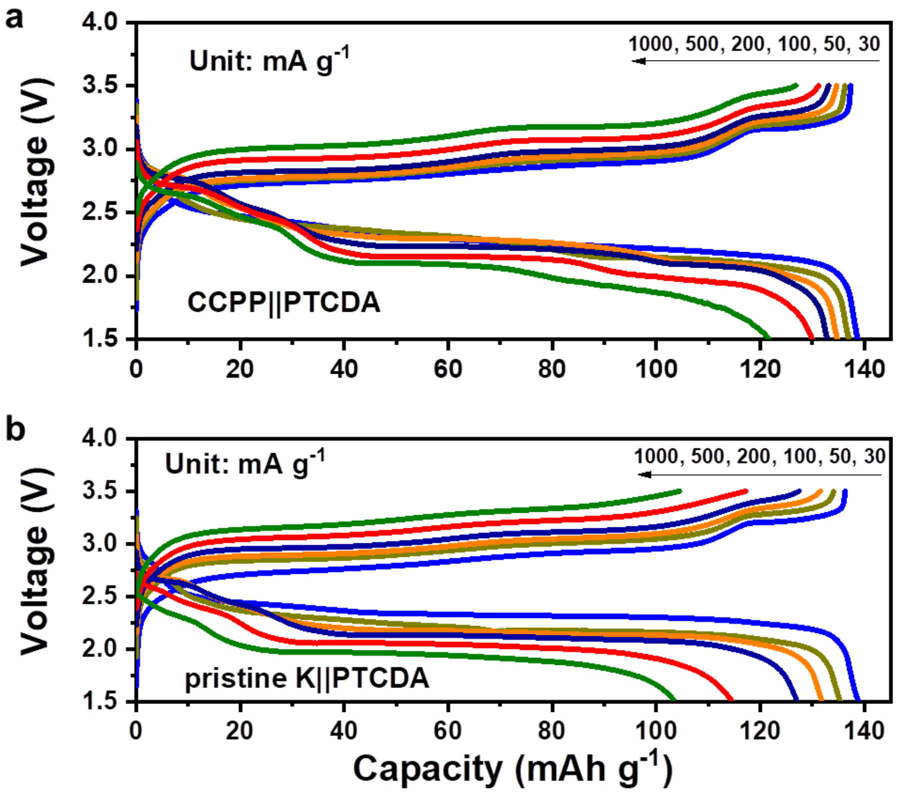

Fig. S28 Charge and discharge profiles of (a) CCPP||PTCDA and (b) pristine K||PTCDA at different current rates. Source data are provided as a Source Data file.

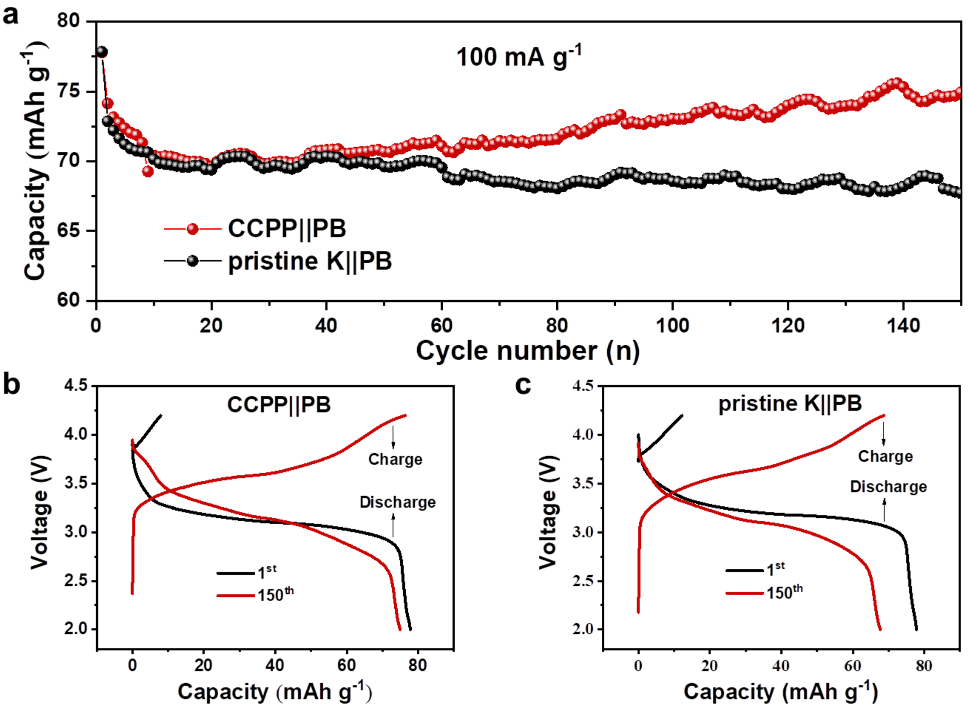

Fig. S29 Electrochemical performances of CCPP||PB and pristine K||PB cells at 100 mA g^{-1} by using KPF_6/EC:DEC electrolyte. (a) Long-term cycling performances. (b) The 1st and 150th charge-discharge profiles of CCPP||PB cells. (c) The 1st and 150th charge-discharge profiles of pristine K||PB cells. Source data are provided as a Source Data file.

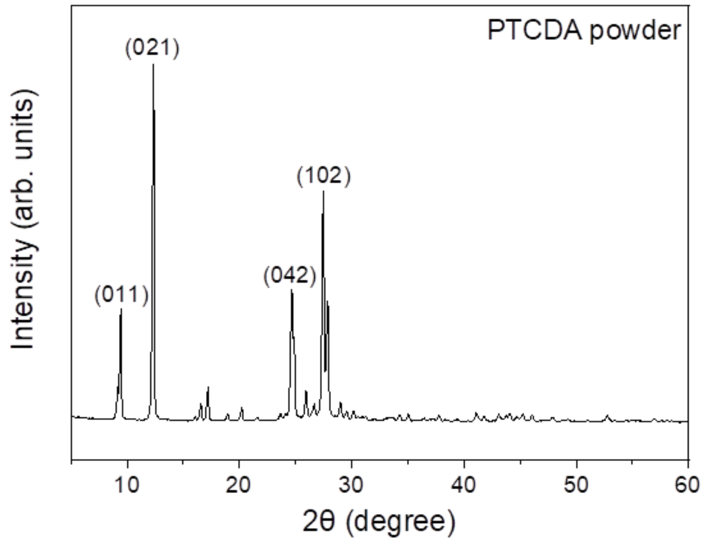

Fig. S30 XRD patterns of PTCDA powder. Source data are provided as a Source Data file.

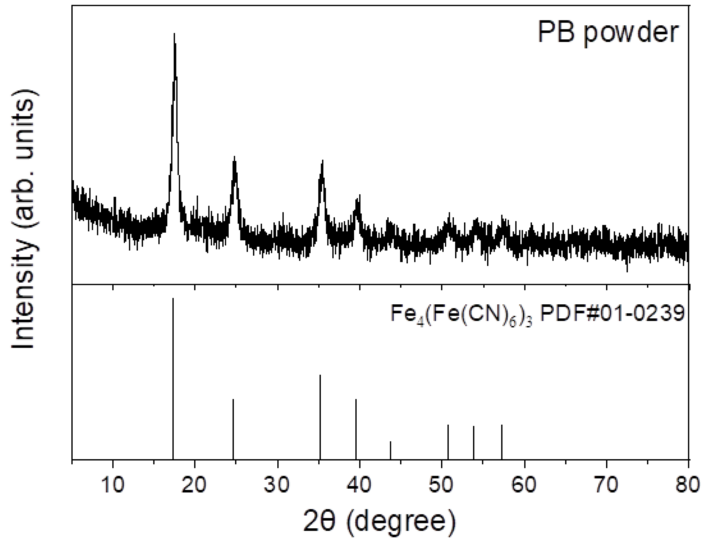

Fig. S31 XRD patterns of Prussian blue powder. Source data are provided as a Source Data file.

Supplementary Tables:

Table S1. The fitting resistance results of symmetric cells for pristine K and CCPP by the equivalent circuit (same as the Figure 3c fitting model) at different cycles.

**Symmetric Cells**	**pristine K**		**CCPP**	
Cycle number	R_s/Ω	R_{in}/Ω	R_s/Ω	R_{in}/Ω
0th	6.214	8337	7.337	2789
10th	5.027	725	6.242	724
50th	——	——	9.447	472
100th	——	——	12.47	373

Table S2. The fitting resistance results of symmetric cells for pristine K and CCPP by the equivalent circuit (same as the Figure S16 fitting model) at different temperatures.

**Temperature**	**0 ºC**		**10 ºC**		**20 ºC**		**30 ºC**		**40 ºC**	
Resistance	R_s/Ω	R_{in}/Ω	R_s/Ω	R_{in}/Ω	R_s/Ω	R_{in}/Ω	R_s/Ω	R_{in}/Ω	R_s/Ω	R_{in}/Ω
pristine K	10.84	84271	9.12	29576	8.015	11100	6.421	4632	7.408	1776
CCPP	11.89	12190	9.978	4932	8.342	2179	9.607	1028	9.766	608
