# Supplementary material for: Tribo-electrochemistry induced artificial solid electrolyte interface by self-catalysis
Source: Nat Commun. 2021 Dec 10;12:7184. doi: 10.1038/s41467-021-27494-z (PMC8664887; doi:10.1038/s41467-021-27494-z)
Supplement: Supplementary file 3 — Description of Additional Supplementary Files [file 41467_2021_27494_MOESM3_ESM.docx]

**1. Description of Additional Supplementary Files (Supplementary Movies)**

**File name:** Supplementary Movie 1

**Description:** Video of explosive reaction and K liquefaction when strong pressure is applied to PTFE and K metal.

**File name:** Supplementary Movie 2

**Description:** Video of drastic action and fire when friction is applied between liquid Na-K alloy and PTFE.

**File name:** Supplementary Movie 3

**Description:** Fire phenomenon when repeatedly lifting and dropping the liquid Na-K alloy droplet onto the PTFE plate.
